# Test–Retest Reliability of Handgrip Strength Measurement in Children and Preadolescents

**DOI:** 10.3390/ijerph17218026

**Published:** 2020-10-31

**Authors:** Jakub S. Gąsior, Mariusz Pawłowski, Piotr J. Jeleń, Eugene A. Rameckers, Craig A. Williams, Robert Makuch, Bożena Werner

**Affiliations:** 1 Department of Pediatric Cardiology and General Pediatrics, Medical University of Warsaw, 02-091 Warsaw, Poland; bozena.werner@wum.edu.pl; 2 Cardiology Clinic of Physiotherapy Division of the 2nd Faculty of Medicine, Bielanski Hospital, Medical University of Warsaw, 01-809 Warsaw, Poland; pawlowskimariusz@o2.pl; 3 Department of Biophysics and Human Physiology, Medical University of Warsaw, 02-004 Warsaw, Poland; piotr.jelen@wum.edu.pl; 4 Department of Rehabilitation Medicine, Research School CAPHRI, Maastricht University, 6229 ER Maastricht, The Netherlands; eaa.rameckers@hetnet.nl; 5 Department of Pediatric Physical Therapy, University for Professionals, AVANSplus, 4818 CP Breda, The Netherlands; 6 Adelante Centre of Expertise in Rehabilitation and Audiology, 6432 CC Hoensbroek, The Netherlands; 7 Faculty of Rehabilitation Science, Pediatric Rehabilitation, Hasselt University, B-3590 Diepenbeek, Belgium; 8 Children’s Health and Exercise Research Centre, St Luke’s Campus, College of Life and Environmental Sciences, Sport and Health Sciences, University of Exeter, Exeter EX1 2LU, UK; c.a.williams@exeter.ac.uk; 9 Department of Physical Education, Kazimierz Pulaski University of Technology and Humanities in Radom, 26-600 Radom, Poland; r.makuch@uthrad.pl

**Keywords:** children, adolescents, muscle strength, grip strength, dynamometer, reliability

## Abstract

The reliability of handgrip strength (HGS) measurement has been confirmed in adults but has been sparsely addressed in pediatric populations. The aims of this study are twofold: to determine whether sex, age and/or hand-dominance influence the test–retest differences and to establish the reliability level of the HGS measurement in typical developing pediatric participants. A total of 338 participants aged 7–13 years were tested using a digital handgrip strength (HGS) dynamometer (Jamar Plus+ Dynamometer) by the same rater on two testing trials separated by a one-day interval between sessions. The HGS testing was conducted according to the American Society of Hand Therapists recommendations. Relative and absolute reliability statistics were calculated. Age influenced the test–retest difference of the HGS measurement as children compared to preadolescents had lower intraclass correlation coefficients (0.95 vs. 0.98), standard error of measurement (SEM) (0.74 vs. 0.78 kg), smallest detectable difference (SDD) (2.05 vs. 2.16 kg) and higher values of the percentage value of SEM (5.48 vs. 3.44%), normalized SDD (15.52 vs. 9.61%) and a mean difference between the test and retest values (0.50 vs. 0.02 kg) for the dominant hand. The results indicate that the protocol using the Jamar digital handgrip dynamometer is a reliable instrument to measure HGS in participants aged 7–13 years with typical development. Clinicians and researchers therefore can have confidence in determining the minimally clinical effect for HGS.

## 1. Introduction

Handgrip strength (HGS) can be easily and quickly measured non-invasively, using portable hand dynamometers [1]. The HGS test is widely applicable in many areas of medicine and sport science to assess isometric muscle strength of the hand and the forearm [2]. Since the HGS is positively correlated with total muscle strength in young healthy subjects, grip strength can be used as an indicator of overall body strength in this population [3]. From the perspective of athletic coaches and healthcare professionals, it is important to evaluate physical fitness qualities using reliable, validated measurements and tools to ensure reproducible results and present meaningful findings [4,5].

In adults, high reliability of the HGS measurement using a Jamar dynamometer has been confirmed in numerous experiments and is considered the gold standard to validate other isometric HGS instruments [1,2,6,7]. A well-established, standardized testing protocol recommended by the American Society of Hand Therapists (ASHT) is commonly utilized in these studies [2,8].

In comparison to adults, the reliability of the HGS measurement has been addressed less frequently in pediatric populations, and with different study methodology (e.g., procedures of the HGS measurement and reliability statistics), participants’ age and used dynamometers [9,10,11,12,13,14,15,16,17,18,19,20,21]. It has been suggested that pediatric participants tend to be more susceptible to several factors that may alter their HGS results and consequently influence the level of reliability of the measurement, e.g., age [13,16], sex-related hand shape [9], hand-dominance [13], testing procedures [11,12] and training status [22]. Test–retest reliability of HGS in large pediatric samples has not been established for the digital handgrip dynamometer when age, sex, and/or hand-dominance are controlled according to testing protocol ASHT recommendations. Therefore, the present study has two aims. The first aim was to determine the reliability of HGS measurements in boys and girls, 7 to 13 years of age, who have met typical developmental milestones. The second aim was to determine if sex, age, or hand dominance affect HGS in participants’ scores.

## 2. Materials and Methods

### 2.1. Participants

A total of 338 participants of both sexes between 7 and 13 years of age voluntarily participated in the study. The participants were divided into two groups based on age: children (7–9 years old) and preadolescents (10–13 years old). This classification is in accordance with other studies where participants younger than 10 years were considered as children and those between 10 and 14 years as preadolescents or early adolescents [23,24,25]. The parents were asked about their children’s training status and known upper-extremity impairments that could influence the HGS. Thirty-two subjects whose parents confirmed regular participation in athletic conditioning (more than twice a week) and any upper-extremity injuries or disorders were excluded from this study. The participants and their parents were instructed that children should refrain from intensive exercise the day before the testing. A written description of the purpose of the study was given to the participants and their parents/legal guardians. Informed written consent and assent forms were obtained from parents/legal guardians and their children, respectively. The study was approved by the University Ethical Committee (KB/120/2015) and followed the rules and principles of the Helsinki Declaration.

### 2.2. Measures

All measurements were performed in a quiet room in a primary school from 8 a.m. to 12 noon. Firstly, the participants were informed about the measurement procedures. Secondly, anthropometric characteristics (body mass, body height) and hand preference were evaluated. The hand preference was assessed in two ways: by asking the participants which hand is used to hold a pen and which hand is used to throw a tennis ball. In the case of discrepancies between the test results, participants were asked to indicate their preferred hand. Before the HGS measurements took place, all participants performed a short warm-up to familiarize themselves with a dynamometer. The familiarization involved grasping the handle, adjusting grip to the handle and performing 2–3 testing trials. The appropriate examination of HGS started 5 min after the familiarization procedure.

The HGS of both hands was measured using the digital handgrip dynamometer—Jamar Plus+ Digital Hand Dynamometer (Patterson Medical, Warrenville, IL, USA). The grip span included five different positions. The second position was used in all participants as previous research has shown this to be the most advantageous position for strength measurements in children [26]. The measurement was conducted according to the standard procedures recommended by the American Society of Hand Therapists (ASHT) [8]. The participants sat upright on a height-adjustable chair with their feet supported. The tested arm was positioned on a table with the shoulders slightly abducted (~10°) and neutrally rotated, the elbow in 90° of flexion, the forearm in 0° between pronation and supination, and the wrist in neutral resting position [8]. The participants were instructed to maintain the position during the test.

Each subject performed three maximum voluntary contractions (tests) for each hand always starting with the dominant hand. The average of the three tests was calculated to two decimal points and used in further analysis. Before each test, the verbal directions were as follows: “This task will measure your grip strength”, then the subjects were asked to squeeze continuously for 3 s on a verbal command: “Squeeze as hard as you can!”. Children were instructed to stop squeezing if they felt pain or discomfort during measurement. The participants were verbally encouraged by the examiner to do their best during the tests. The display of the dynamometer always faced the examiner, thus providing a “blind” measurement to the participants. All HGS measurements were performed by the same researcher. The HGS measurement was repeated the next day under the same conditions and the same time of the day and location.

### 2.3. Statistical Analysis

All values are presented as mean ± SD, unless otherwise stated. Data normality was assessed using the Kolmogorov–Smirnov test. To estimate the effects of hand-dominance, age and sex on possible differences between the test and retest, the repeated-measures analysis of variance (ANOVA) with two within-subject factors: “TIME” (Session 1 versus Session 2), “HAND” (dominant versus non-dominant) and two between-subject factors: “AGE” (7–9 years old—children versus 10–13 years old—preadolescents), “SEX” (boys versus girls) were conducted. The threshold probability of *p* < 0.05 was accepted as the level of significance for all analyses. Statistical calculations were performed using the STATISTICA 10-StatSoft. Inc. software (Tulsa, OK, USA).

Relative and absolute reliability were calculated [27]. To assess relative reliability, intraclass correlation coefficient (ICC) based on a two-way random effects model (absolute agreement, ICC_2.1_) was used [28]. The test–retest reliability is considered to be good when ICC values range from 0.61 to 0.80 and excellent for values between 0.81 and 1.00 [29].

Before calculating absolute reliability, heteroscedasticity was assessed by inspecting Pearson’s correlation coefficients of the absolute difference between the test and retest and the mean of the test and retest [30,31]. If the correlation coefficient (*r*) was between 0 and 0.1, the data were considered as homoscedastic. In such cases, it is recommended that the absolute reliability should be assessed using the standard error of measurement (SEM) [32]. If *r* was greater than 0.1, the data were considered heteroscedastic and consequently the logarithmically transformed coefficient of variation (CV) should be used to assess absolute reliability [31]. The SEM was used to estimate the smallest detectable difference (SDD) also referred to as the “minimum detectable change (MDC)” or “smallest detectable change (SDC)”. To be able to compare reliability of the HGS test with most studies conducted with pediatric participants, percentage value of SEM (SEM%) and normalized smallest detectable difference (nSDD) were also calculated. The SEM was calculated by means of the following equation SEM = SD × (1 − ICC)^0.5^, where SD = the pooled standard deviation of test and retest scores and ICC = calculated intraclass correlation coefficient. The SEM was divided by the mean of the measurements from test 1 and test 2 and multiplied by 100 to give a percentage value (SEM%). The CV was calculated as standard deviation divided by mean and multiplied by 100. The SDD is a linear transformation of the SEM, i.e., 1.96 × $\sqrt{2}$ × SEM [5,28]. The nSDD is the SDD expressed as a percentage of the mean maximum voluntary contraction [13]. The ICC_2.1_ was computed using MedCalc for Windows, version 15.2.2 (MedCalc Software, Inc, Mariakerke, Belgium) [33].

## 3. Results

### 3.1. Participant Characteristics

A total of 306 participants (156 male and 150 female) who met typical developmental milestones between 7 and 13 years of age with mean body height 143.5 ± 13.1 cm, body mass 38.8 ± 12.4 kg and BMI 18.3 ± 3.3 kg∙m^2^ performed HGS tests twice with one-day time period between test and retest. None of the participants felt any pain or discomfort during measurements. A total of 90.2% of the subjects were right-hand dominant and 9.8% were left-hand dominant. Detailed characteristics of the two groups: children (7–9 years old) and preadolescents (10–13 years old) are presented in Table 1.

### 3.2. Effect of Hand Dominance, Age and/or Sex on the Difference between the Test and Retest

The repeated-measures ANOVA revealed statistically significant “AGE” × “TIME” interaction (*F*(1, 302) = 14.51, *p* < 0.001). Since there was no interaction effect between “TIME” and other factors (“HAND”, “SEX”), the reliability statistics were performed for two age-groups: 7–9 years and 10–13 years in boys and girls together only for the dominant hand.

### 3.3. Reliability Assessment

**Figure 1:**
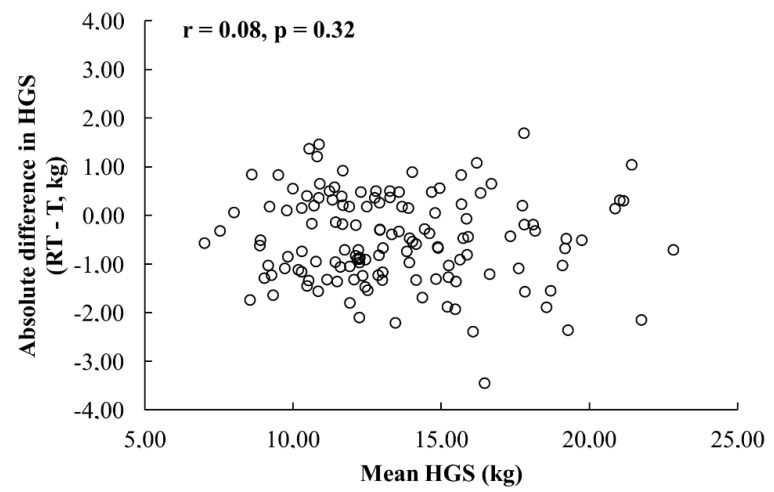
Pearson’s correlation coefficients of the absolute difference between the test and retest and the mean of the test and retest for the dominant hand in 7–9 years age group (T—test, RT—retest).

**Figure 2:**
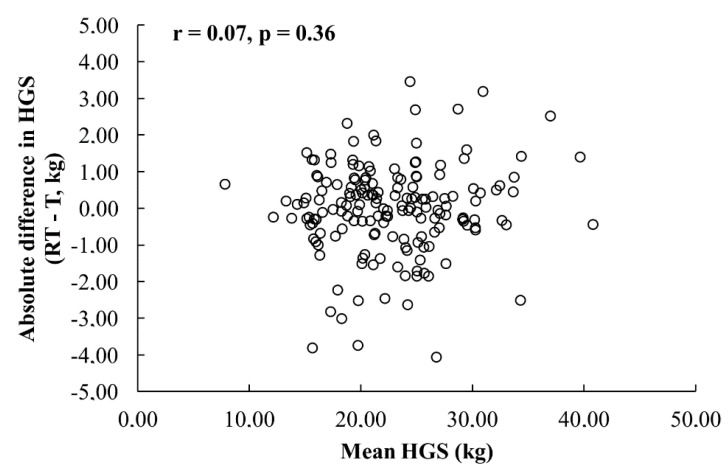
Pearson’s correlation coefficients of the absolute difference between the test and retest and the mean of the test and retest for the dominant hand in 10–13 years age group (T—test, RT—retest).

No presence of heteroscedasticity was observed in children (Figure 1) and preadolescents (Figure 2). Further analysis was conducted using the original data. The ICC_2.1_, SEM and SDD were lower and the SEM%, nSDD and bias were higher for children than preadolescents. The detailed results of the relative and absolute reliability statistics are shown in Table 2.

## 4. Discussion

The results of this study revealed that, of age, sex and hand-dominance, only age influenced the difference between the test and retest of the HGS measures using the Jamar digital hand dynamometer in pediatric participants. For the two age groups and, to the best of our knowledge, for the first time, we report excellent relative reliability, low values of measures of absolute reliability and low bias of HGS measurement in children and preadolescents. None of the participants complained of pain or discomfort during tests indicating feasibility and safety of using this type of dynamometer in a pediatric population.

The reliability in our study was evaluated using commonly established statistical methods used in athletic training, physical therapy and sports medicine [28,32,34]. To test relative reliability, ICC was determined. However, to assess overall reliability, ICC cannot be the sole statistical measure due to the effect of sample heterogeneity [30,34]. As recommended, the results of ICC in conjunction with the absolute measure of reliability, the SEMs were also presented [32,34]. The choice of the SEM was determined as a consequence of the absence of heterogeneity (Figure 1 and Figure 2). SDD (also named MDC or SDC), also calculated, represents the minimal amount of change required to support a change score as real [35]. Presenting all these parameters provides an overall and comprehensive profile of the reliability and allows for better interpretation of the results.

Consideration of the Consensus-based Standards for the selection of health status Measurement Instruments (COSMIN) criteria for reliability study, with an adequate sample size of over 100 participants included in these analyses, allows us to specify this study as excellent [36]. In this experiment, handgrip strength measurement was performed twice in 136 children and 170 preadolescents.

The results obtained in this experiment are in accord with published literature that reported good to excellent (ICC > 0.80) reliability (using Shrout et al. classification [29]) of the HGS measurement using various kinds of the Jamar dynamometer in adults [1,37]. This study is one of several papers concerning reliability of the HGS measurement in children and adolescents with typical development [9,10,11,12,13,14,15,16,17,18,19,20,21]. Due to methodological differences (main aims, procedures of the HGS measurement and reliability statistics), participants’ age and used dynamometers, comparison to all of the mentioned articles is not easily resolved. In the last decade, a research group in Spain published a number of informative review papers concerning test batteries of physical fitness in children and adolescents [18,38,39,40]. These authors indicated that the HGS test could be used to reliably assess musculoskeletal fitness in pediatric participants [18,19,20,21]. However, in these analyzed studies, an analogue dynamometer of a different brand—the TKK dynamometer (Takei, Tokyo, Japan)—was used to measure HGS, and tests were performed using different positioning (standing position with elbow in full extension) [10,15,41] contrary to those proposed in the ASHT recommendations. The TKK dynamometer has the feature that the grip span can be adjusted, whereas other dynamometers, like Jamar, have five fixed positions [18]. Therefore, results of grip strength may vary based on the choice of dynamometer or the measurement protocol [42] but can be confirmed as reliable.

We conducted the HGS measurement twice according to the ASHT procedure recommendations using the Jamar digital handgrip dynamometer with the handlebar in position 2 in participants aged 7–13 years. Taking into account the measurement procedure, the study sample age ranges and the measurement tool used, we can compare our results only to the Molenaar et al. study where the HGS measurement was performed in healthy children in three age groups: 4–6, 7–9 and 10–12 years using, inter alia, an electronic Jamar-like dynamometer (Lode dynamometer) [13]. The results of our study, for both relative and absolute reliability, concur with Molenaar et al. outcomes and show that younger children present lower, but still acceptable reliability of the HGS measurement. Particularly, regarding relative reliability, we noticed excellent test–retest repeatability (ICC ≥ 0.95) in both age groups for the dominant hand. Children obtained a slightly smaller value of ICC than preadolescents but still in the range of excellent reliability (ICC = 0.81 − 1.00). Molenaar et al. revealed that children aged 7–9 years presented good (0.78) and children aged 10–12 years excellent (0.92) test–retest repeatability for the dominant hand [13].

The comparison of statistics calculated using the same formula applied in various studies could be investigated [5]. Our results, similar to those of Molenaar et al. [13], showed that absolute reliability statistics (SEM and SDD) increased and SEM% and nSDD decreased with age, which implies better reliability in older participants. In contrast, we obtained smaller values of the absolute reliability than Molenaar et al., which implies better results of absolute reliability in our study [13]. The differences between our and Molenaar et al. results of reliability statistics may be explained by the different time interval between measurements. In our study the test was repeated the next day in all participants, whereas in the Molenaar et al. study it was a mean of 29 days (range: 3 to 56 days) between the test and retest [13].

Other authors also noted that the reliability of the HGS measurement may depend on children’s age [16]. Svensson et al. showed that Grippit dynamometer can be more reliable in children aged 6 and 14 years than those aged 10 years (lower ICC and higher SEM%) and tried to explain these differences by listing mood, motivation, attention between tests and biomechanical factors as potential influence factors that may decrease reliability in 10-year-old children [16].

Additionally, it was shown that hand-dominance and sex do not affect the difference between the test and retest of the HGS measurements. In the current study, we did not evaluate the influence of handle position and/or hand shape on the reliability of the HGS measurement. However, a few studies primarily investigated the effect of these factors on the maximal HGS in children and adolescents and secondly evaluated whether these factors may affect the reliability of the HGS measurement [9,10,15]. España-Romero et al. [10] and Ruiz et al. [15] stated that, when using the TKK dynamometer, the mathematical equation based on hand span allows the optimal grip span calculation to obtain maximal grip strength in children and teenagers, respectively. Moreover, Ruiz et al. concluded that these equations may improve the reliability of the HGS measurement using this dynamometer [15]. In the present study, we did not assess hand span to calculate optimal grip span and evaluate reliability. We used the second handle position for all participants as we had recently shown that the second handle position of the Jamar digital dynamometer is optimal to measure maximal HGS in a non-athletic healthy pediatric group [26]. The position was also used to establish normative/reference data of handgrip strength [43,44] and to determine and compare the values of the isometric strengths of palmar grip and of the strengths of pulp to pulp, three-point and lateral pinch [45] in healthy participants aged 6 to 19 years. Moreover, Firrell and Crain found that almost 90% of examined children and adults had a maximal grip strength using this handle position irrespective of hand dimensions [46]. In our opinion, the lower reliability of the HGS measurement in younger children observed in our study could not be related to non-optimal handle position.

Authors of studies on normative/reference values for HGS in healthy children have demonstrated that inter alia age and sex have a significant impact on HGS values as evidenced by increasing HGS with age, as well as greater hand strength in boys than girls [47,48,49,50]. Simultaneously to increasing HGS, an age-related increase in hand size in preschool children has also been observed [51], where female hands become narrower than male hands with increasing age [9]. Clerke et al. revealed that square hand shape in female subjects aged 13 to 17 years may result in lower reliability of the HGS measurement using the GripTrack handgrip dynamometer in comparison to long and average hand shape in girls and all kinds of boys’ hand shape [9]. In our study, sex did not influence the test–retest difference of the HGS measurement in pediatric population aged 7–13 years. However, we did not perform the analysis including hand shape as a potential factor. Additional studies will be necessary to verify the potential influence of hand shape on the HGS measurement reliability in childhood using specified dynamometers.

A small increase in HGS values between test and retest was observed particularly in the 7–9-year-old children. The retest measurements were conducted in all participants the next day, therefore changes between the test and retest should not be associated with an improvement of absolute HGS value. A possible learning effect and/or motivational factors may explain the noticed increase in the children’s HGS in our study. A learning effect can be prevented by a larger number of practice trials [32] or by a familiarization procedure [16]. We performed the familiarization with the dynamometer before the HGS measurement. This procedure was the same for all participants, lasting a few minutes, including grasping the handle, adjusting grip to the handle and performing a few test trials. It is possible that such a familiarization might not be enough to acquaint younger children with the equipment.

Some limitations should be mentioned in our study. The participants were divided into two groups based on age in accordance with other studies. These studies grouped participants: those younger than 10 years were considered as children and those between 10 and 14 years as preadolescents or early adolescents, but it is accepted that girls do enter the pre-pubertal phase earlier than boys [52]. Whilst, the division of the groups could be selected according to the maturation level of the participants, e.g., sexual maturation by Tanner stages, we chose not to measure this variable. The invasiveness and the social acceptance of this method by the young participants did not warrant its inclusion in the protocol. The absence of maturation is unlikely to have a significant impact on the main conclusions of the study. We did not include variables such as body mass index (BMI) or fat-free mass (FFT) as potential determinants of changes in HGS between two measurements, i.e., as potential factors that may influence the reliability analysis. It has been shown that, in healthy children, HGS is positively correlated with BMI, body surface area, stature and FFM. Moreover, age-dependent increases in HGS, as well as gender differences, are related to changes of FFM values occurring during childhood [53]. However, like maturational classifications, the influence on age-dependent factors will have little to no influence in the reliability design, because the time in between measurements limited the possibility for personal and environmental factors to change. Finally, the participants were children and preadolescents who met typical developmental milestones. Moreover, the study population was limited to non-athletic children, so caution is needed in applying the results to other pediatric populations, e.g., those in clinical care.

## 5. Conclusions

The results of the present study show that in childhood age influences the difference between the test and retest of the handgrip strength measurement. Preadolescents presented better reliability, whereas children exhibited lower but still acceptable reliability of the HGS measurement using the digital handgrip dynamometer. The present study provides useful information indicating that sports and health professionals measuring hand strength, function and therapy can use this kind of dynamometer as a reliable measurement tool to evaluate isometric handgrip strength in participants aged 7–13 years with normal development and who are non-athletes. Information about the smallest detectable change can help inform researchers and clinicians when interpreting data relative to pediatric participants with chronic medical conditions, as well as sample size calculations for randomized control trial studies.

## Figures and Tables

**Table 1:** Participant characteristics. Values are presented as mean ± SD.

	7–9 years			10–13 years		
	Boys	Girls	Total	Boys	Girls	Total
N	69	68	137	87	82	169
Stature (cm)	133.7 ± 7.6	131.6 ± 8.8	132.6 ± 8.3	152.6 ± 9.4	151.8 ± 8.9	152.2 ± 9.2
Body mass (kg)	31.3 ± 7.8	29.2 ± 6.8	30.3 ± 7.3	45.5 ± 11.7	45.6 ± 11.4	45.6 ± 11.5
BMI (kg∙m^2^)	17.3 ± 2.8	16.7 ± 2.4	17.0 ± 2.6	19.3 ± 3.6	19.6 ± 3.4	19.4 ± 3.5
Dominant hand R/L	63/6	59/9	122/15	81/6	74/8	155/14

N—number of participants; BMI—body mass index; R—right; L—left.

**Table 2:** The handgrip strength for children (7–9 years) and preadolescents (10–13 years) and reliability statistics for dominant hand. Values are presented as mean ± SD.

Age (year)	Test (kg)	Retest (kg)	Difference (kg)	ICC_2.1_ (95% CI)	SEM (kg)	SEM (%)	SDD (kg)	nSDD (%)
7–9	13.25 ± 3.28	13.75 ± 3.34	0.50 ± 0.91	0.95 (0.89–0.97)	0.74	5.48	2.05	15.52
10–13	22.68 ± 5.60	22.70 ± 5.50	0.02 ± 1.23	0.98 (0.97–0.98)	0.78	3.44	2.16	9.61

ICC—intraclass correlation coefficient, SEM—standard error of measurement, SDD—smallest detectable change, nSDD—normalized smallest detectable difference.
